# Female extra‐pair behavior is not associated with reduced paternal care in Thorn‐tailed Rayadito

**DOI:** 10.1002/ece3.7232

**Published:** 2021-03-06

**Authors:** Yanina Poblete, Esteban Botero‐Delgadillo, Pamela Espíndola‐Hernández, Gabriela Südel, Rodrigo A. Vásquez

**Affiliations:** ^1^ Instituto de Ecología y Biodiversidad Departamento de Ciencias Ecológicas Facultad de Ciencias Universidad de Chile Santiago Chile; ^2^ NIAVA: Núcleo de Investigaciones Aplicadas en Ciencias Veterinarias y Agronómicas Instituto de Ciencias Naturales Universidad de Las Américas Santiago Chile; ^3^ Department of Behavioral Ecology and Evolutionary Genetics Max Planck Institute for Ornithology Seewiesen Germany; ^4^ SELVA: Research for conservation in the Neotropics Bogotá Colombia

**Keywords:** *Aphrastura spinicauda*, extra‐pair paternity, facultative male response, nest attendance, parental care, southern Chile

## Abstract

Extra‐pair behavior is present in 76% of socially monogamous bird species with biparental care. This behavior may produce costs to females related to a reduction in paternal care. We estimated the percentage of extra‐pair offspring and quantified paternal care in 44 nests of Thorn‐tailed Rayadito (*Aphrastura spinicauda*) to assess whether males reduce their parental care when females obtain extra‐pair fertilizations. We used data from a sub‐Antarctic population of Rayadito located on Navarino Island (55°4′S, 67°40′W), southern Chile. We found no statistical support for a relationship between variation in paternal care and the percentage of extra‐pair offspring. We discuss how the inability of breeding males to assess their genetic paternity and potential restrictions on behavioral flexibility may explain this result. Additionally, if paternal care is subjected to sexual selection, this could limit a facultative response to female extra‐pair behavior by males. Finally, it is possible that a reduction in paternal care might not have evolved in this particular locality given the low frequency of extra‐pair paternity in our study population.

## Graphical abstract

Thorn‐tailed rayaditos (*Aphrastura spinicauda*), a biparental socially monogamous bird exhibiting extra‐pair offspring, no shows reduced parental care when their social partner obtains extra‐pair fertilizations.

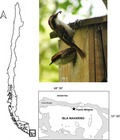

## 1 INTRODUCTION

Extra‐pair behavior is present in ~76% of all socially monogamous birds with biparental care whose genetic mating system has been described through molecular methods (Brouwer & Griffith, 2019). This behavior consists of males and females copulating with individuals other than their social partner, and as a consequence, broods of socially monogamous pairs may have several genetic fathers (Kempenaers & Schlicht, 2010).

From extra‐pair behavior, males can obtain a direct benefit on fitness by increasing the number of sired offspring, while females may obtain indirect benefits by increasing offspring genetic quality (good genes hypothesis, see Hamilton & Zuk, 1982; genetic compatibility hypothesis, see Mitton et al., 1993; Brown, 1997). Additionally, it is possible that unfaithful females reduce the risk of clutch failure in case their social partner is infertile (Kempenaers & Schlicht, 2010; Santema et al., 2020).

Nevertheless, given that males do not provide resources for the extra‐pair female and their offspring, extra‐pair behavior may have some costs that are yet unclear. For instance, in House sparrows (*Passer domesticus*), extra‐pair offspring show lower probability of recruitment into the breeding population and lower lifetime reproductive output compared to within‐pair offspring (Hsu et al., 2014). Moreover, the costs from extra‐pair behavior for females may involve retaliation by the social male that might lead to reduced paternal care when he is not confident on paternity, affecting female reproductive success (e.g., Sheldon & Ellegren, 1998).

An interspecific‐level analysis performed by Birkhead and Møller (1992) reported reduced paternal care levels associated with extra‐pair offspring, however, results from studies at the within‐species level are disparate and far from being conclusive (see e.g., Kempenaers et al., 1998; Sheldon & Ellegren, 1998). For instance, males of species with relatively slow life histories should reduce more strongly their paternal care when females achieve extra‐pair fertilizations than species where adult males have a lower probability to survive to the next breeding event (Wright, 1998). Additionally, it is important to note that regardless of whether females obtain extra‐pair fertilizations, paternal care might vary due to individual differences in age and body condition between males, but also in relation to the fact that a reduction in paternal care may depend on a male's ability to assess paternity (Whittingham & Lifjeld, 1995).

Hence, despite theoretical propositions (Trivers, 1972) along with experimental and correlational approaches have detected reduced paternal care in monogamous birds when females obtain extra‐pair fertilizations (e.g., Sheldon & Ellegren, 1998), within‐species variation in this response is yet to be documented for the vast majority of avian taxa, particularly passerine species from the tropics and the southern hemisphere (Brouwer & Griffith, 2019). It is therefore early to assume reduced paternal care as a widespread cost of extra‐pair behavior for females.

**FIGURE 1:**
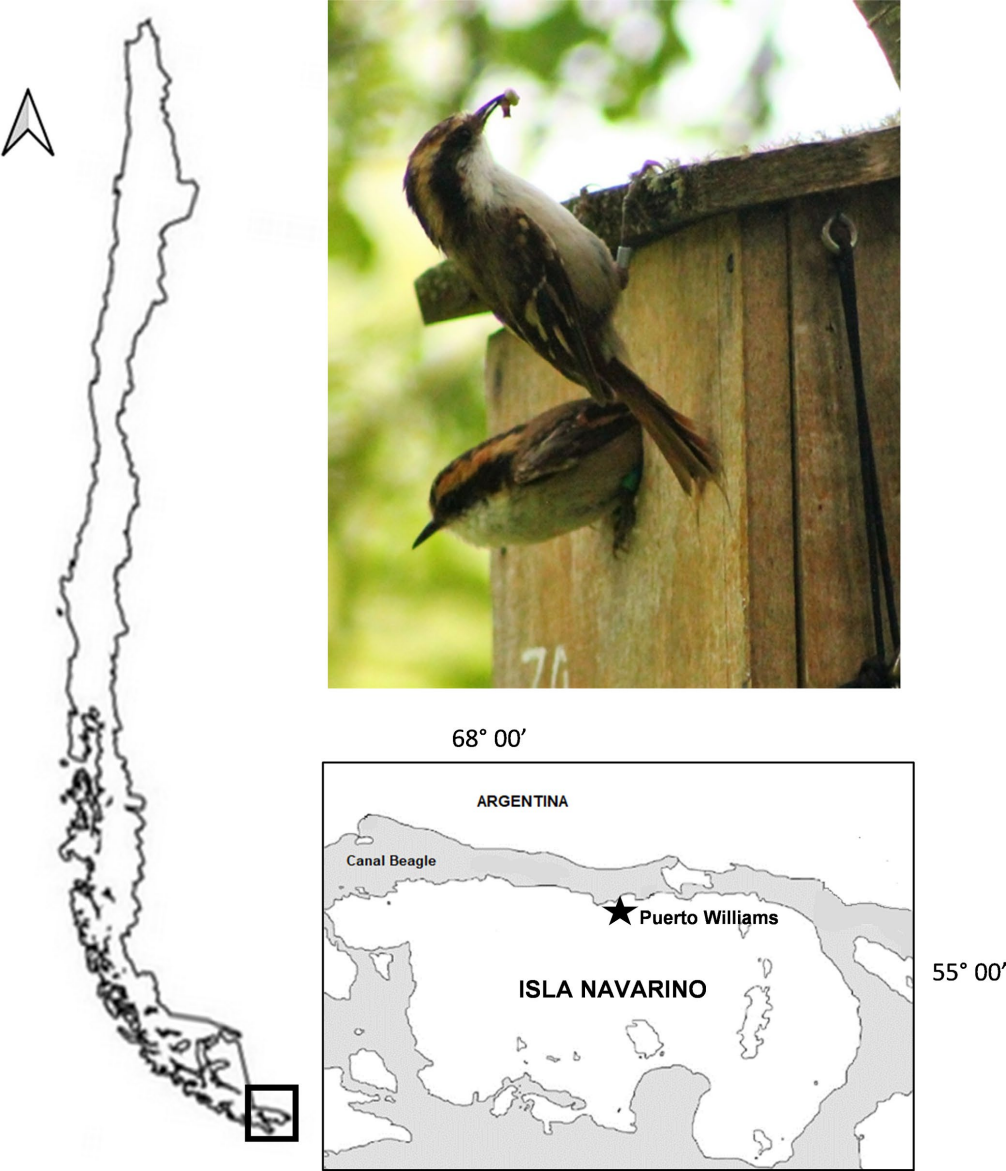
Navarino Island, southern Chile. The black star indicates the location of the nest box plot on the island (3 km west of Puerto Williams). A breeding pair of Thorn‐tailed Rayadito is shown while attending the nest

The Thorn‐tailed Rayadito (*Aphrastura spinicauda*; Figure 1) is a suitable species to assess whether males reduce their parental care when females mate outside the pair‐bond, as it is a biparental socially monogamous bird (Espíndola‐Hernández et al., 2017; Moreno et al., 2005) that exhibits variable rates of extra‐pair offspring across populations (Botero‐Delgadillo, Quirici, Poblete, Ippi, et al., 2020). In this study, we assessed the percentage of extra‐pair offspring and quantify paternal care in nests of Thorn‐tailed Rayadito, in order to assess whether males reduce nest provisioning and attendance when their social partner obtains extra‐pair fertilizations.

## 2 METHODS

### 2.1 Study population and fieldwork

The study was conducted in a sub‐Antarctic population of Thorn‐tailed Rayadito located on Navarino Island (55°4′S, 67°40′W), southern Chile (Figure 1). This locality is dominated by deciduous Magellanic forest that is exposed to strong winds and low temperatures during most of the year (range: −2 to 15.8°C; Pisano, 1977). The breeding season in Navarino Island usually extends from October to January, during the austral spring and summer.

Rayaditos have an average lifespan of 4.8 years, with some individuals reaching the age of 9 years (Quirici et al., 2019). In Navarino, female rayaditos lay one clutch of 1–10 eggs per breeding season, although replacement clutches are observed. Eggs are laid on alternate days, and incubation starts after clutch completion and typically lasts 15–22 days, concluding with the synchronic hatch of the chicks (Moreno et al., 2005). The nestling period ranges from 20 to 23 days (Espíndola‐Hernández et al., 2017).

We collected data from nest boxes during two consecutive breeding seasons, from October to late December 2014 and 2015. We monitored 220 nest boxes that were installed in three large young patches of Magellanic forest, as part of a long‐term study of the breeding biology of Thorn‐tailed Rayadito (Botero‐Delgadillo, Poblete & Vásquez, 2015; Botero‐Delgadillo, Quirici, Poblete, Acevedo, et al., 2020; Botero‐Delgadillo, Quirici, Poblete, Ippi, et al., 2020). Nest box occupation by rayaditos during 2014 and 2015 corresponded to 15% (*n* = 33) and 11.4% (*n* = 25), respectively. Due to strong storms during early spring, five nests failed in 2014 and three in 2015, and thus were not included in our data. Consequently, we recorded a total of 44 breeding pairs (first breeding attempt), seven of which were observed during both years. We recorded a total of 206 nestlings.

We captured breeding adults in their nests 12 days after egg‐hatching, using a manually triggered metal trap. Subsequently, we marked birds with colored plastic bands and individually‐numbered aluminum metal bands. In addition, we measured tarsus length for each parent to the nearest 0.01 cm with a digital caliper, recorded the age as “yearling” or “adult” as appropriate, and the timing of breeding defined as the difference between the first egg date and the date when the first egg in the population was laid. Adults and nestlings were bled (ca. 17 µl) by brachial venipuncture, and blood samples were stored on FTA cards (Whatman^®^) for genetic analysis and molecular sexing.

### 2.2 Parentage analysis

DNA extraction from blood samples and individual genotyping were carried out according to the protocol described in Botero‐Delgadillo et al. (2019). 250 birds (88 adults and 206 nestlings) were sexed using a chromosome‐linked marker (P2/P8) and genotyped at 12 polymorphic microsatellite loci. Parentage analysis was performed in CERVUS 3.0.7 (Kalinowski et al., 2007) using highly informative multilocus genotypes (mean number of alleles per locus: 13.2; combined non‐exclusion probability: 0.00001). Genetic maternity of the social female was confirmed for all nestlings because only three out of 206 offspring mismatched their genetic mother at one locus. These allelic mismatches were used to calculate the frequency of genotyping errors in CERVUS. As outlined by Botero‐Delgadillo et al. (2019), paternity was excluded based on the logarithm‐of‐odds (LOD) score and the critical Delta value (<80%), or whenever there were two or more mismatches between the social male and its putative offspring.

### 2.3 Quantifying paternal care

We positioned a digital video camera (Sony DCR‐68) 3–4 m from the focal nest box to record activity at each nest for 3.5 hr starting between 07:00 and 10:00 hr when nestlings were 17 days old, as this increased the probability of recording parental behavior given that the peak of food demand occurs in the middle stages of nestling development (see Espíndola‐Hernández et al., 2017). The total video footage consisted of 98 hr in 2014 (*n* = 28) and 56 hr in 2015 (*n* = 16), with some nests recorded simultaneously whenever their breeding attempts were synchronyzed. Two members of our research team (PE and GS) played each video footage in the laboratory to compile information on parental behavior using the SMPlayer^©^ (version 0.8.0). Another member (YP) replayed the videos independently and double‐checked the data. During video checking, the observers were blind toward the hypothesis being tested (i.e., no knowledge on the presence of extra‐pair young in an any given nest). We then calculated provisioning and nest cleaning rates, measured as the total number of provisioning and nest cleaning visits (i.e., removing of fecal sacs) per number of 17 days old nestling for each nest.

### 2.4 Statistical analysis

We used linear mixed‐effects models (LMM) with a Gaussian error distribution and log‐link function to evaluate the relationship between the percentage of extra‐pair offspring and paternal care for 44 sampled nests. We used separate models that included male provisioning and nest cleaning rates as dependent variables. Timing of breeding (*z*‐scores calculated using the mean value and standard deviation for each year) was included as covariate. The social father/mother and nest box identities were included as random intercepts. Between‐season variation in the male provisioning and nest cleaning rates was accounted for by calculating *z*‐scores using the mean value and standard deviation for each year. Although male age (“yearling” or “adult”) and body size (measured as *z*‐scores of tarsus length) may relate to variation in parental behavior, as well as the time at which video recording started, a preliminary analysis did not evidence an effect of these variables on male provisioning and nest cleaning rates (see Tables S1 and S2). We thus did not include these variables in the analysis so as to not overparameterize the models. For all analyses, we checked and validated model assumptions. We considered an effect to be statistically supported when the 95% CI around the estimate did not overlap zero.

## 3 RESULTS

**FIGURE 2:**
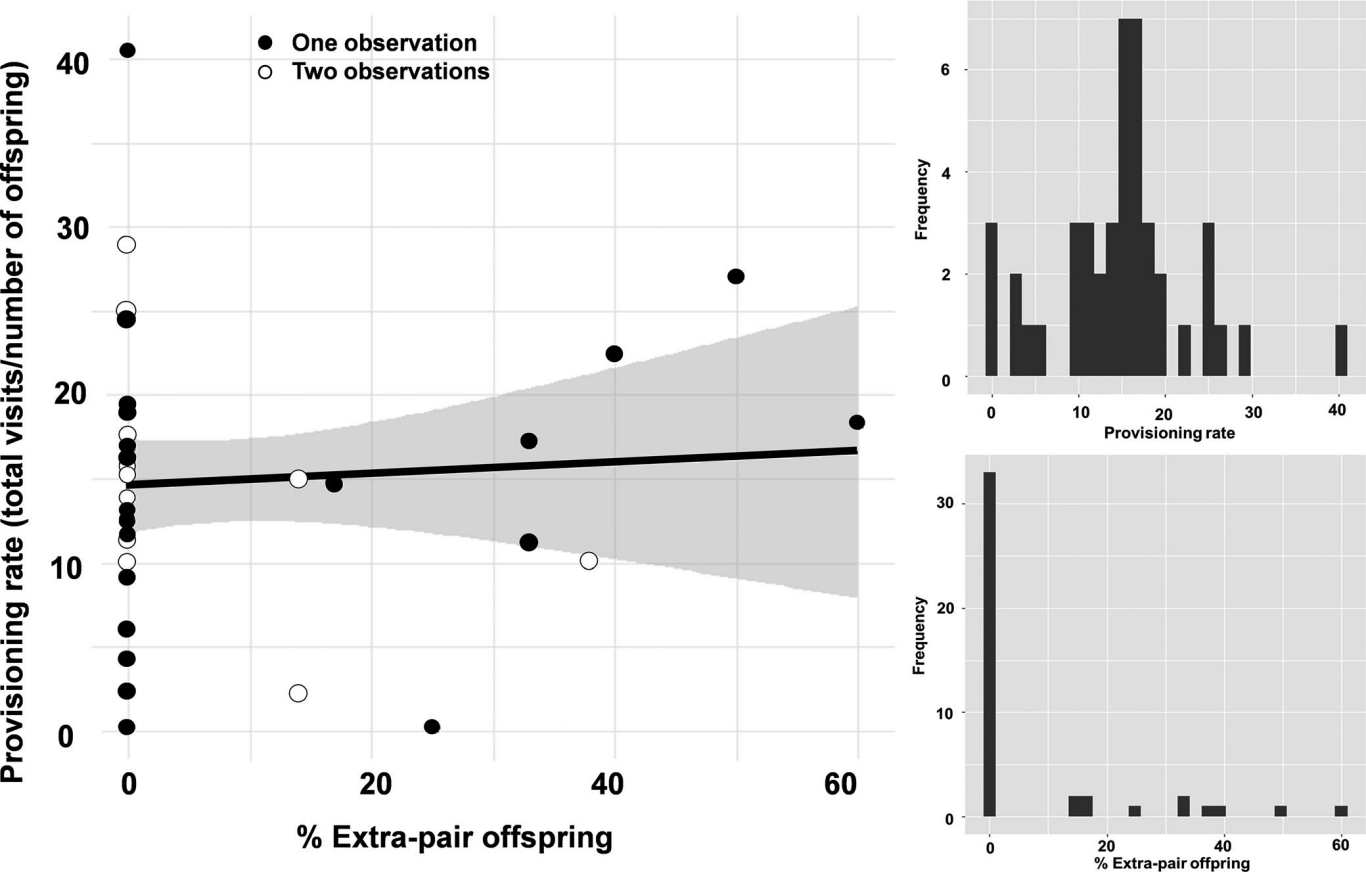
Relationship between provisioning rates (during 3.5 hr) and the percentage of extra‐pair offspring in 44 nests of Thorn‐tailed Rayadito (males with one observation, *n* = 30; males with two observations, *n* = 7). Shown are the raw data (points) and model predictions with 95% confidence intervals (lines and shaded area). Histograms showing the observed variation in provisioning rates and percentage of extra‐pair offspring in the population

**FIGURE 3:**
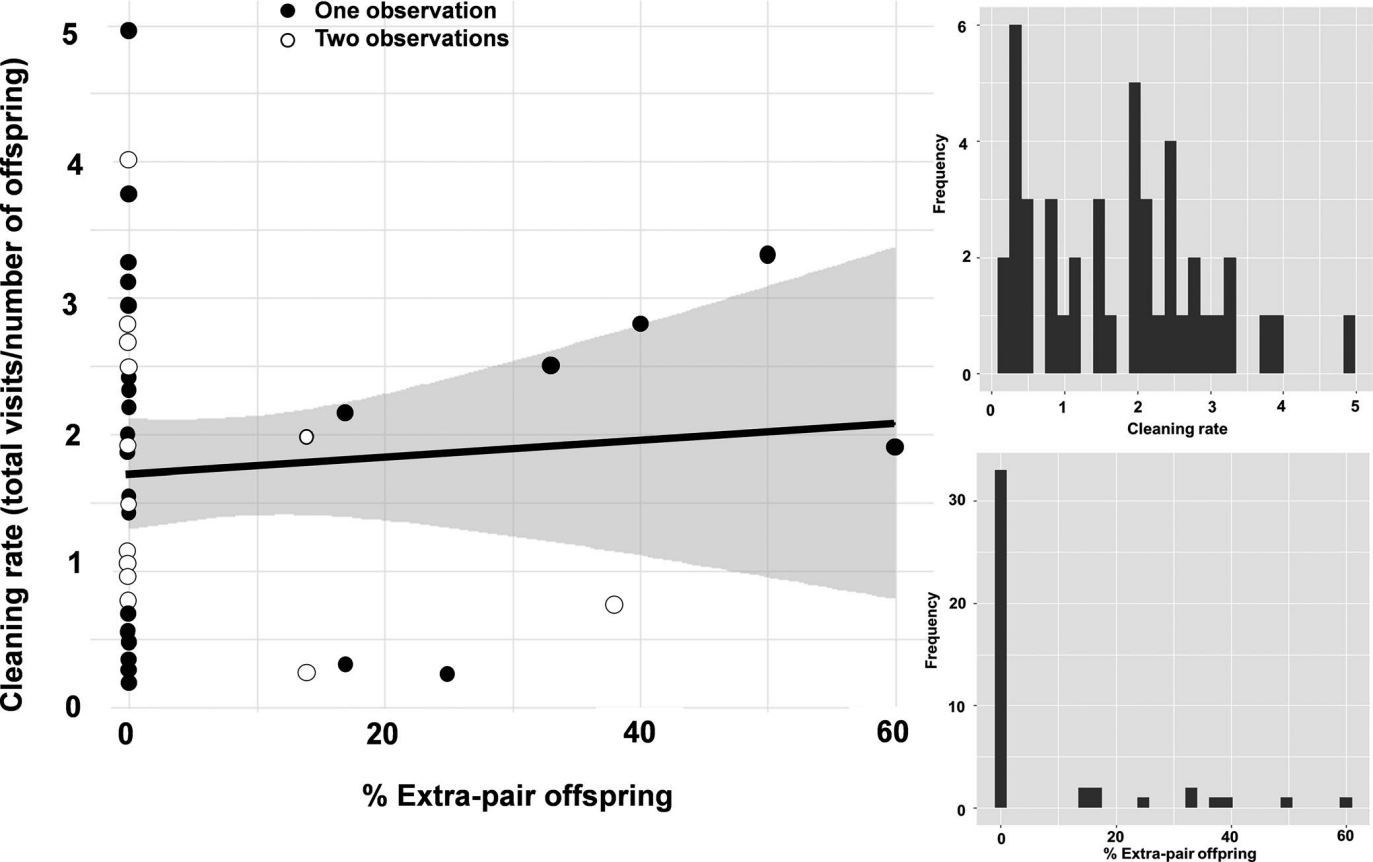
Relationship between nest cleaning rates (during 3.5 hr) and the percentage ofextra‐pair offspring in 44 nests of Thorn‐tailed Rayadito (males with one observation, *n* = 30; males with two observations, *n* = 7). Shown are the raw data (points) and model predictions with 95% confidence intervals (lines and shaded area). Histograms showing the observed variation in nest cleaning rates and percentage of extra‐pair offspring in the population

Eleven of the 44 broods that were genotyped (25%) had at least one extra‐pair young (range: 1–3). Seventeen of the 206 nestlings (8.3%) were extra‐pair offspring. The percentage of extra‐pair offspring varied from 0% to 60% (Figures 2 and 3). All nests with extra‐pair offspring had only one additional genetic father. Male rayaditos made on average 14.9 ± 7.9 provisioning visits to their nest during the 3.5 hr of observation (Figure 2), and made 1.8 ± 1.2 nest cleaning visits (Figure 3). Evidence of polygyny or males providing parental care at both primary and secondary nests was not observed during this study. We found no statistical support for a relationship between the percentage of extra‐pair offspring and male provisioning (Table 1; Figure 2) or nest cleaning rates (Table 2; Figure 3).

**TABLE 1:** Results from a mixed‐linear model showing the effect of extra‐pair offspring percentage (% EPO) on male provisioning rates in a population of Thorn‐tailed Rayadito (*n* = 37 individuals; 30 captured once and seven captured twice; 44 total observations)

	Estimate	SE	L 95% CI	U 95% CI
Intercept	−4.08e^−02^	1.02e^−02^	**−0.063**	**−0.020**
% EPO	2.27e^−04^	3.93e^−04^	−0.001	0.001
Timing of breeding	−1.18e^−05^	7.72e^−04^	−0.002	0.002
**Random effect**	***σ*^2^**			
Social father identity	0.0000			
Social mother identity	0.0003			
Nest box	0.0002			
Residual	0.0009			

Note

Timing of breeding was included as covariate in the model. The father and mother social identities and nest‐boxes were included as random effects. *Z‐*scores were calculated for the response variable and timing of breeding using the mean value and standard deviation for each year. *SE*: standard error; L/U 95% CI = lower/upper bound for the 95% confidence interval. Bold numbers indicate intervals that did not include zero.

**TABLE 2:** Results from a mixed‐linear model showing the effect of extra‐pair offspring percentage (% EPO) on male cleaning rates in Thorn‐tailed Rayadito (*n* = 37 individuals; 30 captured once and seven captured twice; 44 total observations)

	Estimate	*SE*	L 95% CI	U 95% CI
Intercept	−6.43e^−03^	1.55e^−03^	**−0.009**	**−0.002**
% EPO	3.84e^−05^	5.93e^−05^	−0.000	0.000
Timing of breeding	−4.73e^−05^	1.12e^−04^	−0.000	0.000
**Random effect**	***σ*^2^**			
Social father identity	4.233e^−13^			
Social mother identity	1.440e^−05^			
Nest box	1.903e^−05^			
Residual	1.588e^−06^			

Note

Timing of breeding was included as covariate in the model. The father and mother social identities and nest‐boxes were included as random effects. *Z‐*scores were calculated for the response variable and timing of breeding using the mean value and standard deviation for each year. *SE*: standard error; L/U 95% CI = lower/upper bound for the 95% confidence interval. Bold numbers indicate intervals that did not include zero.

## 4 DISCUSSION

We found no evidence for a link between variation in paternal care and the percentage of extra‐pair offspring in broods of Thorn‐tailed Rayadito in Navarino Island. This suggests that male rayaditos might not adjust parental care levels when females obtain extra‐pair fertilizations and, hence, that unfaithful females would not suffer the costs associated with partner retaliation in the form of reduced paternal care.

Several factors may explain our results. First, a reduction of paternal care would depend on the ability of males to guard their mates and detect his partner while engaging in extra‐pair behavior, but this may be difficult in highly forested areas such as Navarino (see Biagolini et al., 2017). Moreover, a reduction in parental care would also depend on a male's ability to confidently determine paternity (Whittingham & Lifjeld, 1995), but there is no information on the existence of mechanisms to discriminate extra‐pair offspring in rayaditos.

Second, parental behavior in rayaditos could be functionally integrated with other traits, as for instance aggressive individuals could exhibit reduced provisioning rates (see Barnett et al., 2012), which in turn would reduce behavioral flexibility (Wagner & Stadler, 2003). This could explain, at least partially, why we failed to find evidence for an association between variation in paternal care levels and the percentage of extra‐pair offspring.

Third, parental behavior may be a sexually selected trait, and hence, poor paternal care could lead to lower male fitness regardless of whether females have the potential to mate with other males in the population (Wagner et al., 1996). Additionally, theoretical models suggest that in socially monogamous species, such as Thorn‐tailed Rayadito, males may reduce parental care only below a certain threshold of paternity confidence (Whittingham et al., 1992).

It is important to note that reduced paternal care may also depend on the frequency of extra‐pair behavior in the population. Theoretically, a high percentage of unfaithful females may lead both to a reduction in paternal care benefits and an increase in the probability of extra‐pair mating for males. This would ultimately favor a reduction in paternal care (see McNamara et al., 2002). A long‐term study of extra‐pair paternity in rayaditos showed that yearly rates of extra‐pair offspring in Navarino are low (usually < 10%; Botero‐Delgadillo, Quirici, Poblete, Ippi, et al., 2020), and thus a reduction of paternal care as a response to female extra‐pair behavior might not have evolved in this population.

In summary, our data do not support the idea that male rayaditos adjust their parental care in response to female unfaithfulness, but this requires further confirmation. Longer‐term monitoring and further studies in other populations that include diverse paternal care measurements, will provide greater insight into the relationship between female extra‐pair behavior and male facultative responses in this and other bird species.

## CONFLICT OF INTEREST

The authors declare that they have no conflict of interest.

## AUTHOR CONTRIBUTIONS

**Yanina Poblete:** Conceptualization (lead); data curation (lead); formal analysis (equal); funding acquisition (supporting); investigation (equal); methodology (equal); resources (equal); writing – original draft (lead); writing – review and editing (equal). **Esteban Botero‐Delgadillo:** Formal analysis (equal); funding acquisition (supporting); investigation (equal); methodology (equal); resources (equal); writing – review and editing (equal). **Pamela Espíndola‐Hernández:** Formal analysis (supporting); investigation (equal); methodology (equal). **Gabriela Südel:** Formal analysis (supporting); methodology (equal). **Rodrigo A. Vásquez:** Funding acquisition (lead); investigation (equal); project administration (lead); resources (equal); supervision (lead); writing – review and editing (supporting).

## Supporting information

Supplementary Material

## Data Availability

The datasets and R code used during the current study are available from the Dryad Digital Repository (https://doi.org/10.5061/dryad.hhmgqnkg3).
