# Supplementary material for: Female extra‐pair behavior is not associated with reduced paternal care in Thorn‐tailed Rayadito
Source: Ecol Evol. 2021 Mar 6;11(7):3065–71. doi: 10.1002/ece3.7232 (PMC8019036; doi:10.1002/ece3.7232)
Supplement: Supplementary file 1 — Supplementary Material [file ECE3-11-3065-s001.docx]

**Female extra-pair behavior is not associated with reduced paternal care in Thorn-tailed Rayadito**

Yanina Poblete, Esteban Botero-Delgadillo, Pamela Espíndola-Hernández, Gabriela Südel and Rodrigo A. Vásquez

**Table S1.** Results from linear model showing the effect of body size (tarsus length), age (‘yearling’ or ‘adult’) and timing of start recording on provisioning rate in Thorn-tailed Rayaditos males (*n* = 37 individuals; 30 captured once and 7 captured twice; 44 total observations). *Z-*scores were calculated for the response variable using the mean value and standard deviation for each year. SE: standard error; L/U 95% CI = lower/upper bound for the 95% confidence interval. Bold numbers indicate intervals that did not include zero.

* Parameter estimates and SE (standard errors) were estimated relative to ‘yearling’ level in variable age.

|  | **Estimate** | **SE** | **L 95% CI** | **U 95% CI** |
| --- | --- | --- | --- | --- |
| Intercept | -0.06 | 0.01 | -0.09 | -0.03 |
| Body size | -0.01 | 0.01 | -0.02 | 0.01 |
| Age* | 0.01 | 0.01 | -0.01 | 0.04 |
| Timing of start recording | 0.00 | 0.00 | -0.00 | 0.00 |

**Table S2.** Results from linear model showing the effect of body size (measured as tarsus length), age (‘yearling’ or ‘adult’) and timing of start recording on cleaning rate in Thorn-tailed Rayaditos males (*n* = 37 individuals; 30 captured once and 7 captured twice; 44 total observations). *Z-*scores were calculated for the response variable using the mean value and standard deviation for each year. SE: standard error; L/U 95% CI = lower/upper bound for the 95% confidence interval. Bold numbers indicate intervals that did not include zero.

* Parameter estimates and SE (standard errors) were estimated relative to ‘yearling’ level in variable age.

|  | **Estimate** | **SE** | **L 95% CI** | **U 95% CI** |
| --- | --- | --- | --- | --- |
| Intercept | -9.47e^-3^ | 2.13e^-3^ | **-1.37e-02** | **-5.17e^-3^** |
| Body size | -4.85e^-4^ | 8.92e^-4^ | -2.29e^-3^ | 1.31e^-3^ |
| Age* | 1.45e^-3^ | 1.81e^-3^ | -2.19e^-3^ | 5.10e^-3^ |
| Timing of start recording | 2.83e^-5^ | 1.88e^-5^ | -9.67e^-5^ | 6.63e^-6^ |
